# Development and Validation of an Interpretable Machine Learning Prediction Model for Total Pathological Complete Response after Neoadjuvant Chemotherapy in Locally Advanced Breast Cancer: Multicenter Retrospective Analysis

**DOI:** 10.7150/jca.97190

**Published:** 2024-08-01

**Authors:** Ziran Zhang, Bo Cao, Jinghua Wu, Chengtian Feng

**Affiliations:** Department of Breast Diseases, Jiaxing Women and Children's Hospital, Wenzhou Medical University, Jiaxing, Zhejiang, 314000, P.R. China.

**Keywords:** Locally advanced breast cancer, Neoadjuvant chemotherapy, Pathological complete response, Machine learning, Predictive model, SHapley additive exPlanations

## Abstract

**Objective:** This study aims to develop an interpretable machine learning (ML) model to accurately predict the probability of achieving total pathological complete response (tpCR) in patients with locally advanced breast cancer (LABC) following neoadjuvant chemotherapy (NAC).

**Methods:** This multi-center retrospective study included pre-NAC clinical pathology data from 698 LABC patients. Post-operative pathological outcomes divided patients into tpCR and non-tpCR groups. Data from 586 patients at Shanghai Ruijin Hospital were randomly assigned to a training set (80%) and a test set (20%). In comparison, data from our hospital's remaining 112 patients were used for external validation. Variable selection was performed using the Least Absolute Shrinkage and Selection Operator (LASSO) regression analysis. Predictive models were constructed using six ML algorithms: decision trees, K-nearest neighbors (KNN), support vector machine, light gradient boosting machine, and extreme gradient boosting. Model efficacy was assessed through various metrics, including receiver operating characteristic (ROC) curves, precision-recall (PR) curves, confusion matrices, calibration plots, and decision curve analysis (DCA). The best-performing model was selected by comparing the performance of different algorithms. Moreover, variable relevance was ranked using the SHapley Additive exPlanations (SHAP) technique to improve the interpretability of the model and solve the "black box" problem.

**Results:** A total of 191 patients (32.59%) achieved tpCR following NAC. Through LASSO regression analysis, five variables were identified as predictive factors for model construction, including tumor size, Ki-67, molecular subtype, targeted therapy, and chemotherapy regimen. The KNN model outperformed the other five classifier algorithms, achieving area under the curve (AUC) values of 0.847 (95% CI: 0.809-0.883) in the training set, 0.763 (95% CI: 0.670-0.856) in the test set, and 0.665 (95% CI: 0.555-0.776) in the external validation set. DCA demonstrated that the KNN model yielded the highest net advantage through a wide range of threshold probabilities in both the training and test sets. Furthermore, the analysis of the KNN model utilizing SHAP technology demonstrated that targeted therapy is the most crucial factor in predicting tpCR.

**Conclusion:** An ML prediction model using clinical and pathological data collected before NAC was developed and verified. This model accurately predicted the probability of achieving a tpCR in patients with LABC after receiving NAC. SHAP technology enhanced the interpretability of the model and assisted in clinical decision-making and therapy optimization.

## Introduction

Breast cancer (BC) constitutes the most prevalent cancer among women worldwide and is a leading cause of cancer-related mortality in this demographic 1. Although there has been a growing public awareness of BC and substantial progress in detection technologies that have improved the rates of early detection, around 6-7% of patients in China till receive a diagnosis of locally advanced breast cancer (LABC) at the time of diagnosis 2. LABC typically refers to BC with significant involvement of the lymph nodes in the axillary region without evidence of distant metastasis during diagnosis 3. This stage of BC presents particular challenges due to the difficulty of surgical removal, high risk of distant metastasis, and poor prognosis, leading to a relatively high clinical mortality rate. Therefore, relying entirely on surgical treatment frequently leads to suboptimal disease control. This underscores the critical importance of integrated treatment strategies in improving patient outcomes. Neoadjuvant chemotherapy (NAC) serves as the standard preoperative treatment for patients with LABC 4. It helps to lower the stage of the cancer as well as optimize surgical plans, leading to higher success rates for breast-conserving surgeries and sentinel lymph node biopsies. NAC also allows targeted treatments based on the tumor's response to chemotherapy. The achievement of total pathological complete response (tpCR) is a critical indicator of NAC efficacy, with patients attaining tpCR demonstrating significantly better overall and disease-free survival than those who do not achieve tpCR. However, currently, only approximately 20-40% of patients reach tpCR post-NAC 5-7, and chemotherapy can induce adverse reactions such as bone marrow suppression, hepatic and renal impairment, and cardiac failure in some patients 8, 9. Therefore, it is imperative to accurately predict the effectiveness of chemotherapy at an early stage to maximize patient benefit and minimize the potential negative consequences linked to NAC.

Presently, clinical assessment methods for NAC efficacy primarily include macroscopic observation of morphological changes in the tumor during NAC and microscopic evaluation of tumor cell numbers post-NAC 10, 11. Utilizing pre-NAC clinical-pathological data to predict NAC efficacy could facilitate the timely adjustment of treatment plans and develop personalized strategies to improve patient outcomes. This could also increase the chances of achieving a tpCR and ultimately improve the prognosis for patients. Consequently, this study aimed to develop a machine learning (ML) model based on pre-NAC clinical pathology data to predict the probability of achieving tpCR in LABC patients following NAC. SHapley Additive exPlanations (SHAP) technology was incorporated to provide intuitive explanations of the ML model's predictions and assist clinicians in devising more personalized diagnostic and treatment plans.

## Materials and Methods

### Patients selected and study designed

**Figure 1:**
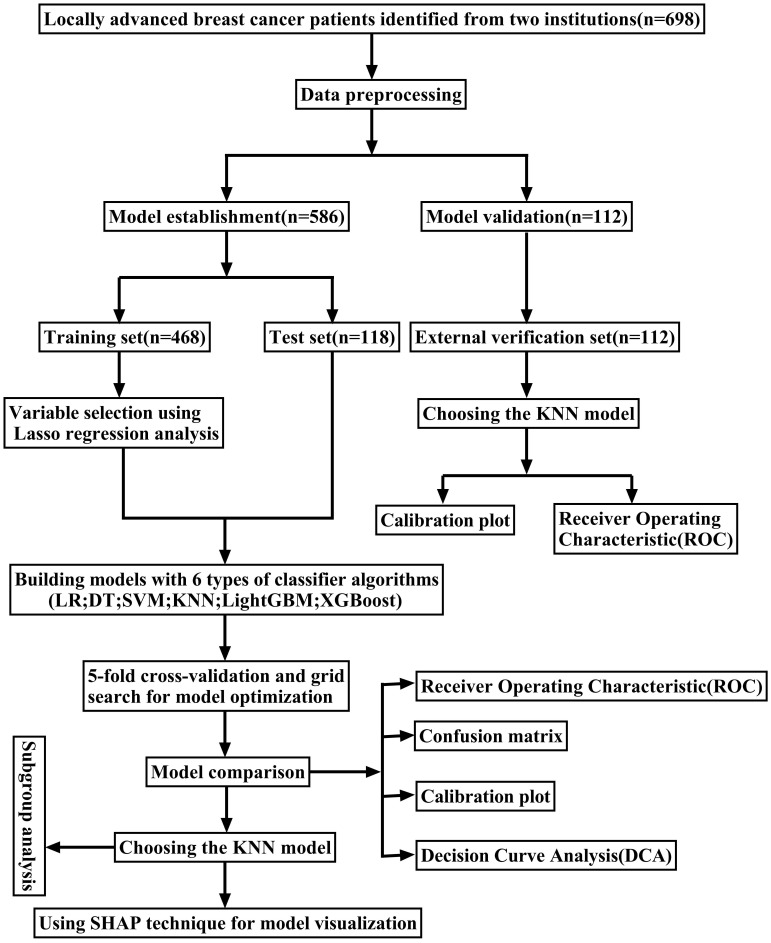
The flowchart of the study.

In this retrospective study, data from 586 patients with LABC who underwent NAC treatment at the Ruijin Hospital, affiliated with Shanghai Jiao Tong University, between May 1, 2014, and December 31, 2021, were selected to construct an ML model. Moreover, data from 112 LABC patients treated with NAC at Jiaxing Women and Children's Hospital from January 1, 2016, to January 31, 2023, were utilized for external validation of the optimal ML model (Figure 1).

### Inclusion and exclusion criteria

Inclusion criteria for this study were as follows: (1) Diagnosis of unilateral primary invasive BC confirmed via core needle biopsy; (2) Cytological verification of axillary lymph node metastasis; (3) Completion of NAC and radical surgical treatment; (4) Availability of comprehensive clinical and pathological data. Exclusion criteria included: (1) Male patients; (2) Incomplete clinical and pathological records; (3) Patients who were intolerant to NAC; (4) Presence of distant metastases during NAC; (5) Patients with recurrent or bilateral BC.

### Treatment regimen

The administration of chemotherapy regimens and courses to patients strictly adhered to the guidelines prescribed by the Chinese Society of Clinical Oncology for the specific year. All participants completed at least four cycles of NAC, primarily comprising taxane- and/or anthracycline-based chemotherapy drugs, with some regimens also integrating platinum-based drugs. Additionally, a subset of patients with HER-2-positive status received targeted therapy with trastuzumab and/or pertuzumab. To evaluate the effectiveness of the treatment, ultrasonographic examinations were performed after every two cycles. Within four weeks following the completion of the last chemotherapeutic cycle, patients had surgical procedures, which involved either mastectomy or breast-conserving surgery, along with axillary lymph node dissection.

### Pathological efficacy assessment

The pathological response was determined based on the histological evaluation of the surgical specimens. A tpCR is defined as the absence of all invasive cancer cells in the breast tissue and axillary lymph nodes, irrespective of residual ductal carcinoma *in situ* (ypT0/isN0) 5, 12.

### Data collection

**Figure 2:**
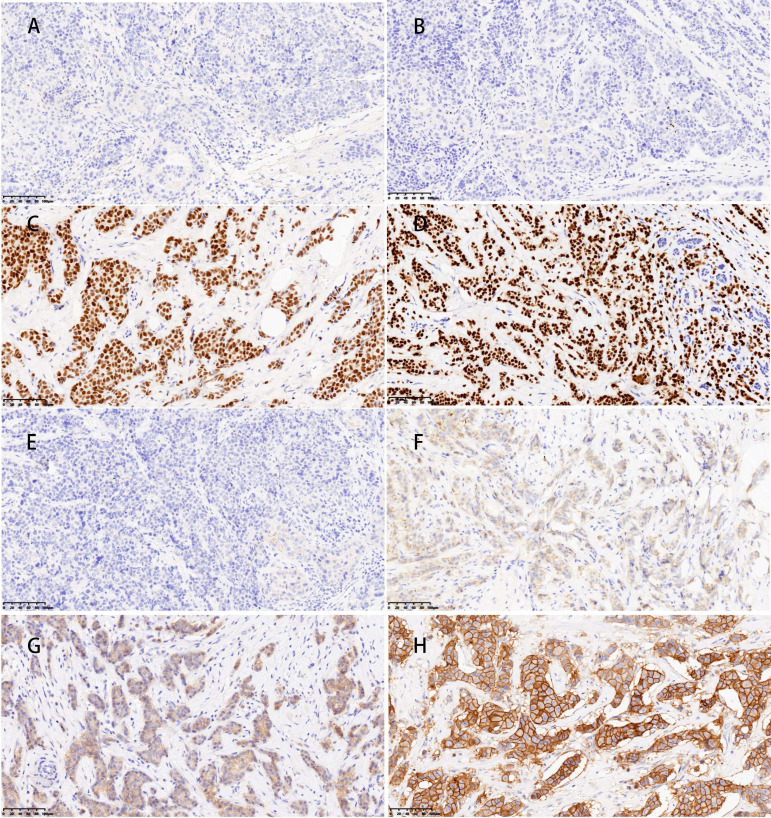
**Representative images of ER, PR, and HER-2 by immunohistochemical staining.** ER (A) and PR (B) negative expression in the nucleus. ER (C) and PR (D) showed strong positive in the nucleus; E.HER-2(0) (no staining or incomplete and faint/barely perceptible membrane staining in ≤10% of tumor cells); F HER-2(+) (incomplete and faint/barely perceptible membrane staining in >10% of tumor cells); G HER-2(++) (weak/moderate complete membrane staining in>10% of tumor cells or complete and intense membrane staining in ≤10% of tumor cells); H HER-2(+++) (complete and intense membrane staining in>10% of tumor cells); Magnification (X20), Scale bars = 100 µm.

This study extracted the following clinical data from patient records: demographic characteristics including age, body mass index (BMI), and menopausal status; tumor attributes such as size, multifocality, axillary lymph node fusion, and histological grade; as well as the status of molecular markers, including estrogen receptor (ER), progesterone receptor (PR), human epidermal growth factor receptor-2 (HER-2), and Ki-67 expression. Furthermore, the study examined the molecular subtypes and post-operative pathological stages. The BMI was computed by dividing the weight at diagnosis (in kilograms) by the square of the height (in meters). The size of the tumor was assessed by ultrasonography. Multifocality refers to the presence of two or more tumor foci within the breast. Histological grading was categorized into grade III and others. The Immunohistochemical (IHC) test for ER and PR uses a positivity threshold of ≥ 1% expression (Figure 2 A-D). This means that patients with ER/PR expression of ≥ 1% are classified as hormone receptor (HR) positive 13. The status of HER-2 was initially evaluated through IHC, with (+++) indicating positivity, 0 or (+) indicating negativity, and (++) requiring further assessment by fluorescence *in situ* hybridization (FISH) to determine HER-2 gene amplification (Figure 2 E-H) 14. BC molecular subtyping was simplified into three categories: triple-negative BC (TNBC) (HR (-), HER-2 (-)), HER-2 positive (BC) (HR (-)/HR (+), HER-2 (+)), and Luminal (BC) (HR (+), HER-2 (-)). This classification aided in understanding the therapeutic response of different BC subtypes.

### Statistical analysis

This study conducted data processing and analysis using Statistical Package for the Social Sciences (SPSS) software (version 26) and R programming language (version 4.3.2). The receiver operating characteristic (ROC) curves were utilized for continuous variables to determine the optimal cutoff points, thereby converting these continuous variables into binary data, which were then presented as frequencies (%). Comparisons between categorical data groups were performed using the Pearson chi-square test. The Least Absolute Shrinkage and Selection Operator (LASSO) regression analysis was employed to identify independent predictive factors closely associated with tpCR. To evaluate the presence of multicollinearity among the predictive factors, the variance inflation factor (VIF) was computed for each variable. A VIF value below 5 indicated the absence of significant multicollinearity 15. Six distinct ML predictive models were developed, and their performance was evaluated using a variety of metrics, including ROC curves, precision-recall (PR) curves, and confusion matrices. Calibration plots were used to compare the calibration capabilities of different models, and decision curve analysis (DCA) was applied to evaluate the clinical utility of the models. Furthermore, SHAP technology analyzed the contribution of each predictive factor to the model's predictive outcomes, thereby enhancing the interpretability of the models. All statistical analyses were conducted using two-tailed tests, with a *p*-value of less than 0.05 considered statistically significant.

## Results

### Selection of cutoff values for continuous data

ROC analysis demonstrated statistically significant differences between the ROC curves for tumor size and Ki-67 expression (***p*** < 0.05), indicating that these variables had discriminative predictive value. However, no significant differences were observed in the ROC curves for age and BMI (***p*** > 0.05), indicating a lack of predictive differentiation based on these factors alone. The Youden Index was employed at points of maximum differentiation on the ROC curves for variables with significant differences to establish optimal cutoff values for these variables. This approach facilitated the bifurcation of continuous data into binary categories: tumor size was divided at a threshold of 3.14 cm (≤ 3.14 cm and > 3.14 cm), and Ki-67 expression was categorized at 47.5% (< 47.5% and ≥ 47.5%). Variables that did not show significant variations in their ROC curves were divided into two categories based on their median values: age was categorized into ≤ 50 years and > 50 years, and BMI was divided at 23.52 (≤ 23.52 and > 23.52).

### Baseline characteristics

This study encompassed a total of 698 patients, with ages ranging from 21 to 89 years. Of these, 586 were recruited from Ruijin Hospital, affiliated with Shanghai Jiao Tong University, and randomly allocated to the training and test sets in an 8:2 ratio. Subsequently, the remaining 112 patients from Jiaxing Women and Children's Hospital constituted the external validation set. Overall, the distribution of variables across the datasets was fundamentally consistent, with the exception of age, chemotherapy regimen, and Ki-67 levels, which exhibited statistically significant differences (P<0.05) as shown in Table 1. Based on the outcomes related to tpCR, patients were further categorized as tpCR and non-tpCR groups. The tpCR rates observed in the training, test, and external validation set were 27.6%, 28.0%, and 25.9%, respectively. Significant statistical differences (p < 0.05) were found in the training set for variables like tumor size, Ki-67, molecular subtype, targeted therapy, and chemotherapy regimen. These findings form a crucial foundation for selecting variables to further develop predictive models. Similar significant differences in molecular subtype, targeted therapy, and chemotherapy regimen (p < 0.05) were also demonstrated in the test set, further validating the importance of these variables in the model. Concurrently, the external validation set highlighted significant differences in Ki-67, molecular subtype, and chemotherapy regimen indicators (p < 0.05), underscoring the clinical applicability of these variables (Table 2).

### Feature selection

**Figure 3:**
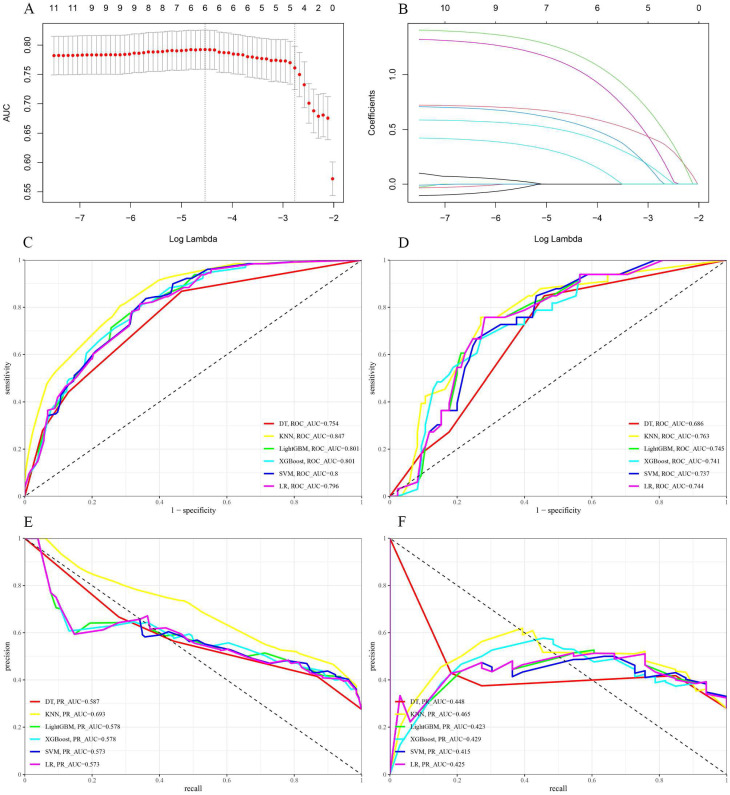
(A and B) LASSO regression model. A delineates selecting the most appropriate regularization parameter, λ, employing a ten-fold cross-validation approach within the LASSO regression framework. B showcases a coefficient profile plot, which is constructed based on the sequence of log (λ) values, providing insights into the behavior of the model's coefficients across different values of λ. C and D display the receiver operating characteristic (ROC) curves for six distinct models within the training and test sets, respectively. E and F present these models' precision-recall (PR) curves, comparing their performance in both the training and test sets.

The LASSO regression utilizes a penalization strategy to effectively compress variables, resulting in regression coefficients of certain variables being driven to zero. This process enhances variable selection and simplification by retaining only those variables with non-zero coefficients, achieving efficient variable selection and dimensionality reduction. The study accurately found the most optimal value of the regularization parameter, λ, by employing ten-fold cross-validation 16, 17. More precisely, vertical reference lines were marked at the minimum value of λ (λ = 0.010) and one standard error above this minimum value (λ = 0.062), as displayed in Figure 3A. At a log (λ) value of -2.766, five key variables with non-zero coefficients were identified: tumor size, Ki-67, molecular subtype, targeted therapy, and chemotherapy regimen. These variables have been demonstrated to significantly influence the model's predictive capability, as shown in Figure 3B. This methodology reduced the model's complexity, and the most influential variables were preserved, thereby enhancing the model's predictive accuracy and interpretability.

### Multicollinearity test

Upon conducting multicollinearity tests on the five predictive variables obtained, it was observed that the tolerance values for tumor size, Ki-67, molecular subtype, targeted therapy, and chemotherapy regimen were all greater than 0.1 (0.988, 0.877, 0.805, 0.952, and 0.903). Moreover, the VIF were all below 5 (1.012, 1.140, 1.243, 1.050, and 1.108). Therefore, it can be concluded that there was no significant multicollinearity among these variables. Hence, each provides unique and independent information for the prediction model.

### Development and evaluation of machine learning predictive models

In this research, six ML predictive models were developed, including logistic regression (LR), decision tree (DT), support vector machine (SVM), K-nearest neighbors (KNN), light gradient boosting machine (LightGBM), and extreme gradient boosting (XGBoost) models, to predict the probability of achieving a tpCR in patients with LABC following NAC. In order to enhance the performance of the model, a resampling strategy called five-fold cross-validation was used, along with a technique called grid search to identify the optimal hyperparameters 18.

Model efficacy was assessed primarily through the comparison of ROC curves, PR curves, and confusion matrices. The training set yielded an area under the curve (AUC) of 0.847 (95% CI: 0.810-0.883) for the KNN model and 0.801 (95% CI: 0.760-0.842) for the XGBoost model (Figure 3C). The DeLong test revealed a statistically significant difference in the AUC values between these two algorithms (***p*** = 0.001). In the test set, the AUC values for the KNN and LightGBM models were 0.763 (95% CI: 0.670-0.856) and 0.745 (95% CI: 0.653-0.836), respectively (Figure 3D). Based on the DeLong test, there was no statistically significant difference in the AUC values of these two algorithms (***p*** = 0.561). Due to the unequal distribution of outcome events in the dataset, relying exclusively on AUC metrics may not provide a comprehensive assessment of model performance. Thus, PR curves were employed to overcome this constraint, providing a more thorough evaluation of the model's performance. The KNN model exhibited PR values of 0.693 and 0.465, signifying a higher average precision compared to other models, as depicted in Figures 3E and F. Furthermore, in the training set, the confusion matrix for the KNN model showed an accuracy rate of 0.741, an F1 score of 0.632, and a kappa coefficient of 0.447, which was the highest observed accuracy rate. Furthermore, the confusion matrix of the KNN model used to analyze the test set showed significantly high metrics. The accuracy rate was 0.712, the F1 score was 0.595, and the kappa coefficient was 0.387, as displayed in Table 3. Based on these results, the KNN model was found to be the most suitable in terms of its predictive accuracy and stability. Model calibration was also a critical aspect of the evaluation process. Calibration plots and Brier scores were utilized to measure the discrepancy between model predictions and actual events 19.

**Figure 4:**
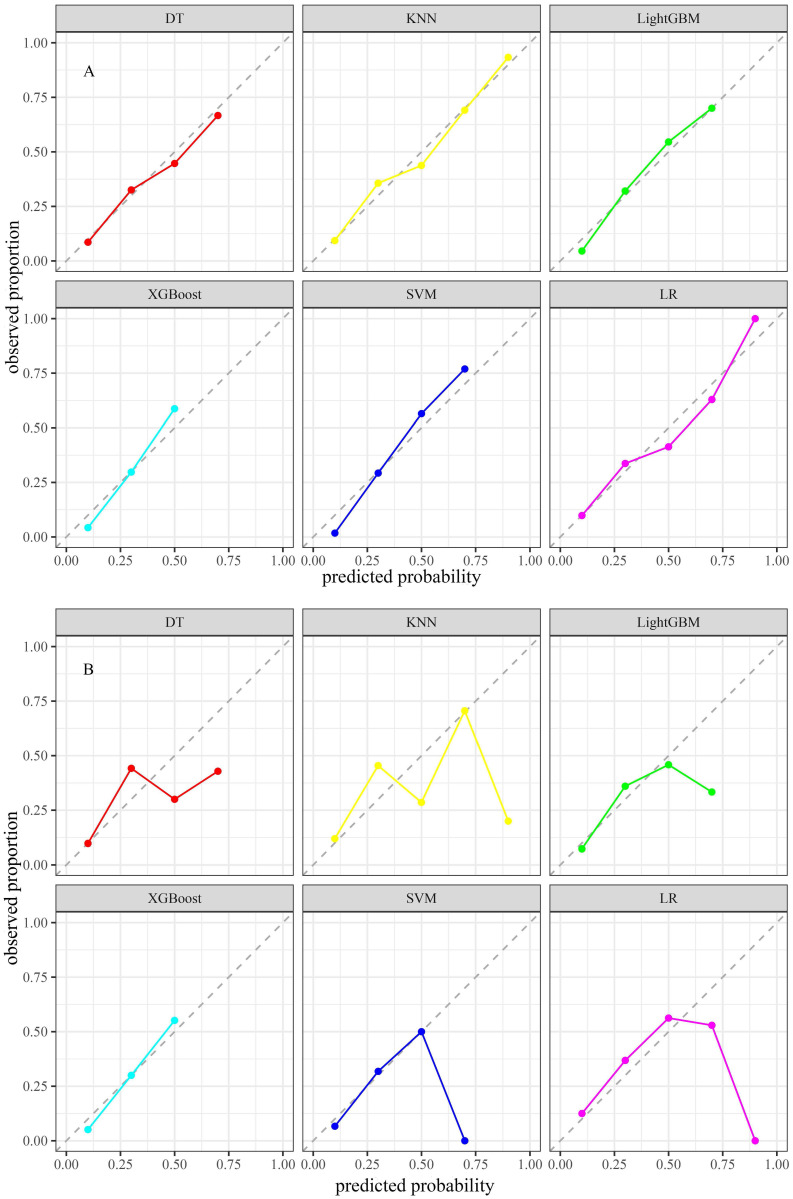
Calibration plots of six models in the training set (A) and the test set (B).

**Figure 5:**
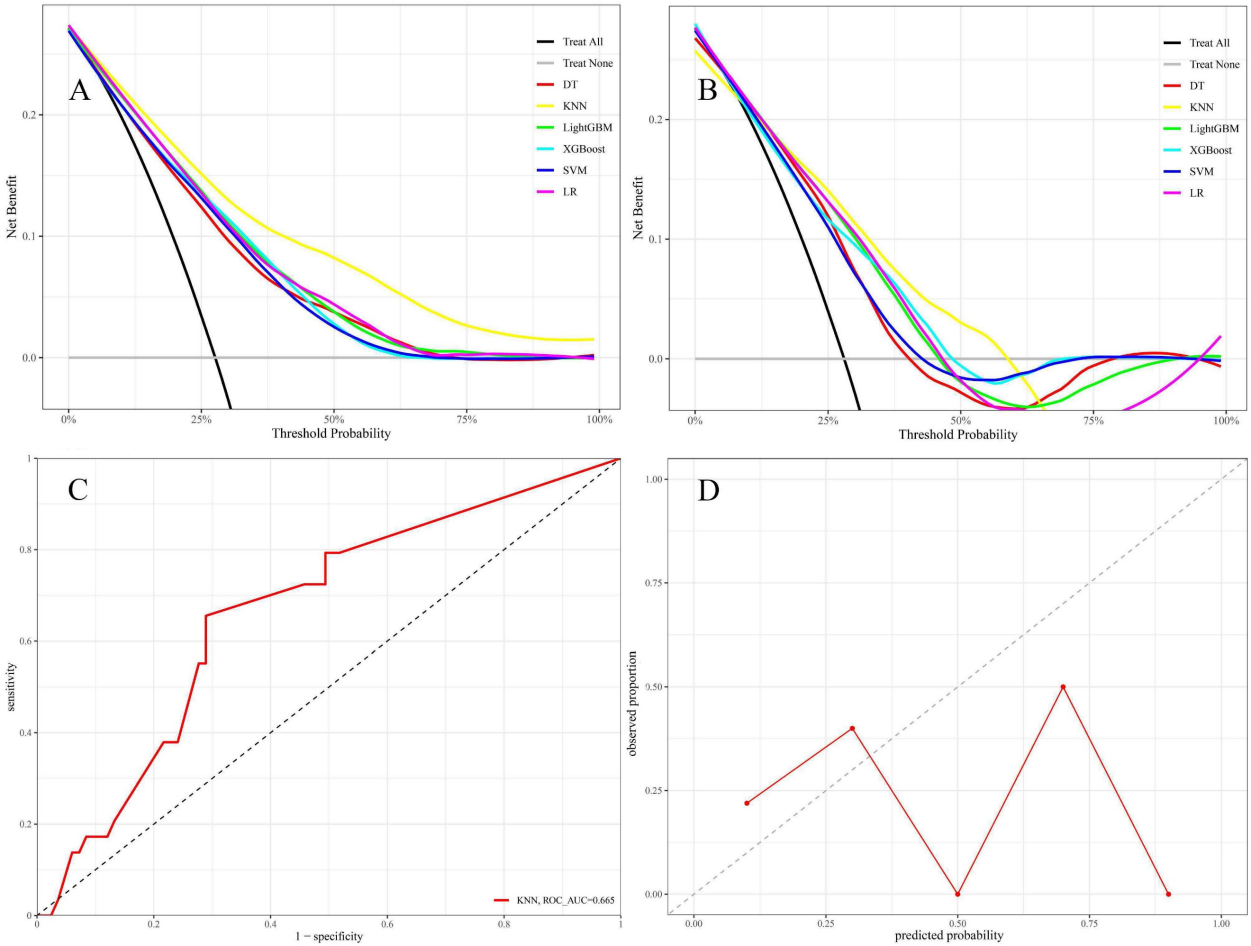
The DCA was conducted for six models in both the training set (A) and test set (B) using different threshold probabilities; in the DCA plots, the bottom gray line represents the scenario where no patients achieved tpCR following NAC, while the black diagonal line represents the scenario where all patients achieved tpCR after NAC. The x-axis of DCA represents the threshold probability, and the y-axis represents the net benefit after subtracting the disadvantages. Theoretically, the further the DCA curve is from these two extreme lines, the higher the net clinical benefit of the model. The external validation set contains the AUC (C) and calibration plot (D).

The KNN model exhibited Brier scores of 0.135 and 0.182 in the training and test sets, respectively, which were significantly below the generally accepted threshold of 0.25, indicating excellent calibration (Figures 4A and B). The DCA assessed the net advantage across several threshold probabilities to determine the clinical usefulness of the model. The findings from the DCA indicated that all six ML models performed better than the default strategy for most threshold ranges. Among these models, the KNN model showed the most significant overall advantage in terms of net benefit, as observed in both the training and test sets (Figures 5A and B). Consequently, the KNN model was selected as the final predictive model. This model offers a dependable tool for clinical practice by efficiently diagnosing the likelihood of obtaining tpCR in LABC patients post-NAC. To further confirm the external applicability of the selected model, an independent dataset from Jiaxing Women and Children's Hospital was employed for external validation. The results indicated that the model achieved a high AUC value of 0.665 (95% CI: 0.555-0.776), accompanied by a low Brier score of 0.220 in the external validation set, as demonstrated in Figure 5C and D. Moreover, the model displayed the capability to predict breast pathological complete remission (bpCR), with AUC values of 0.828 (95% CI: 0.790-0.866) and 0.791 (95% CI: 0.708-0.875) in the training and test sets, respectively.

### Interpretability of machine learning models

**Figure 6:**
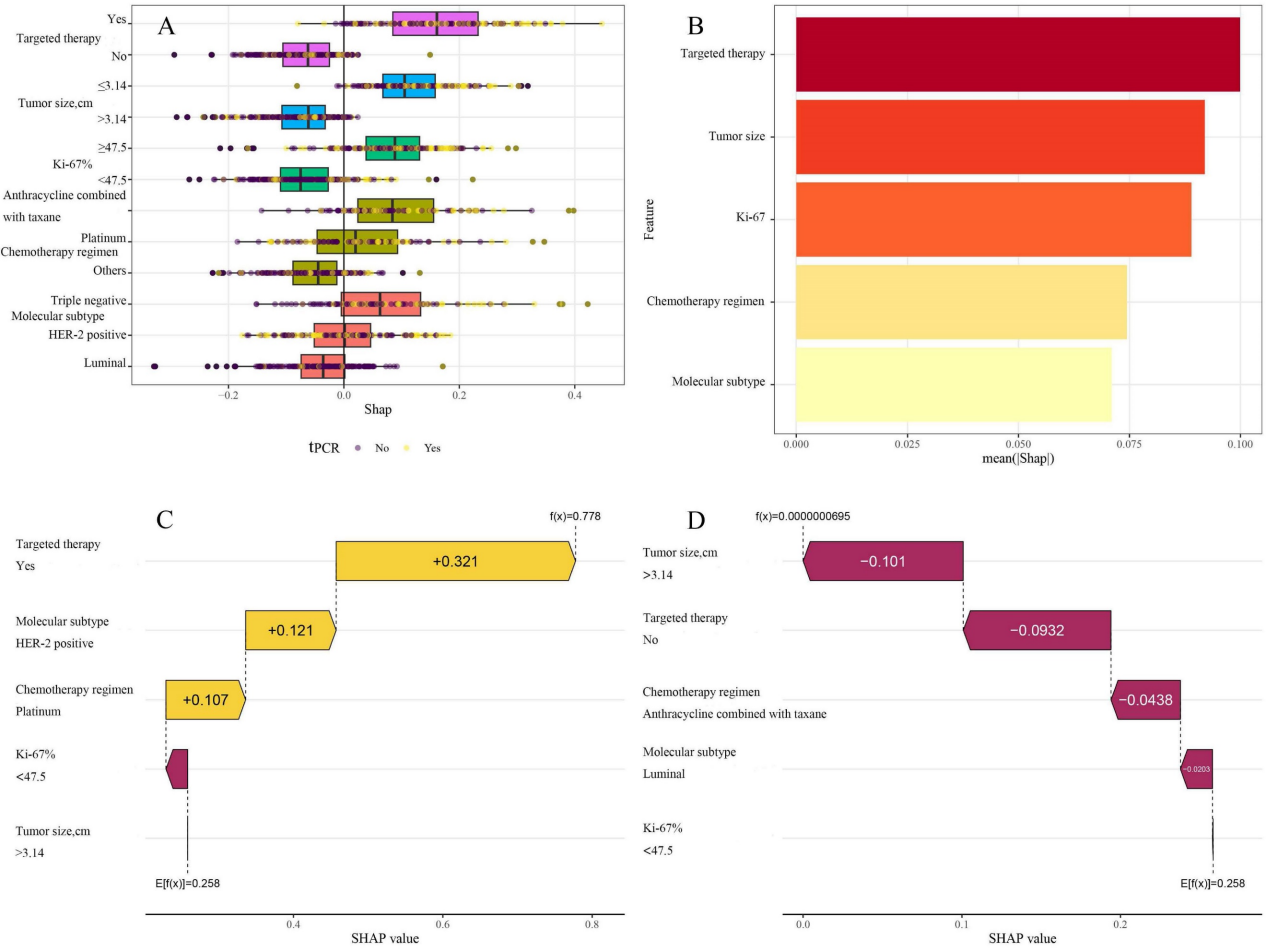
A: Aggregate SHAP values for categorical variables; B: Feature importance plot; in the feature importance plot, each predictive variable corresponds to a line segment, the length of which indicates the weight of the variable's impact on tpCR. A longer line signifies a higher weight, reflecting the variable's importance in predicting tpCR. C: A patient's waterfall plot demonstrating tpCR; D: Individual waterfall plot for a patient who did not achieve tpCR. E [f(x)] represents the baseline prediction probability; f(x) denotes the model's final prediction probability for a given input.

ML models are often perceived as "black boxes" due to the opacity of their internal mechanisms, which can limit clinicians' trust in the model outcomes. The Shapley values of game theory have given rise to SHAP technology, which intends to tackle this challenge. This methodology offers a straightforward and efficient means of elucidating the predicted results of models, exposing the connection between variables and model estimations, and calculating the magnitude and direction of each variable's influence on the results. The decision-making process of models is transparent because of the visualization techniques utilized by SHAP, which makes it suitable for interpretive analysis across a variety of ML models 20. To gain a deeper understanding of the contribution of different variables within the KNN model to the predictive outcomes, the SHAP values of each variable were calculated. They were displayed in descending order of importance, thus visually demonstrating the extent of each variable's impact on the predictions (Figure 6A and B). To validate the interpretability of the model, two typical samples were selected: one non-tpCR patient and one tpCR patient, and each presented their SHAP value waterfall graphs separately (Figures 6C and D). The predictive score for the tpCR patient was significantly higher (0.778) compared to the non-tpCR patient (< 0.001). Examining these individual waterfall plots made it understandable how each variable influences the model's final prediction and the interactions between different variables. This visualization technique enhanced the transparency of the model's decision-making process, thereby bolstering clinicians' confidence in the model outcomes.

## Discussion

Given the ongoing progress in medical technology and changes in treatment philosophies, NAC has emerged as a standard therapeutic approach for patients with LABC. The assessment of clinical response in patients receiving NAC treatment predominantly depends on the RECIST 1.1 criteria, which evaluate tumor size alterations 21. However, this method often fails to yield satisfactory outcomes. Recently, there has been a strong focus on researching clinical or molecular biomarkers that can adequately predict the effectiveness of NAC. Various possible predictive indications, including clinical, pathological, radiological, and molecular biology features, have been found 7, 15, 22-25. Comprehensive testing is not feasible for every patient due to economic costs and operational complexities. ML, a pivotal branch of artificial intelligence, can process and analyze vast amounts of high-dimensional complex data, uncovering nonlinear relationships and subtle factors that traditional methods may fail to detect. ML facilitates more precise feature identification and selection by mitigating subjective biases among observers. In recent years, the application of ML in medical research has significantly increased, especially in disease prediction, where it has shown remarkable advantages. Currently, various ML models have been developed to predict pCR following NAC for BC. Among these, magnetic resonance imaging (MRI) is considered the most sensitive imaging technique for assessing and predicting NAC outcomes, and several ML models based on MRI have been developed 26-29. However, these studies are still in their early stages, with relatively small patient cohorts, limiting their statistical power. Additionally, the high cost and complexity of MRI limit its widespread use. In addition to imaging features, clinical and pathological characteristics can also be used to predict NAC response. For instance, Kim and Meti et al. have developed ML models based on clinical and pathological features to predict pCR after NAC for BC 7, 30. However, these studies have several limitations, including the inclusion of some early-stage breast cancer patients in their samples, a lack of external validation, and insufficient model interpretability. Accordingly, this study focused on patients with LABC, a group with a poor prognosis, and employed readily available clinical pathology data as key predictive factors. The objective was to develop a simple, reliable, and highly interpretable ML predictive model to accurately forecast the early probability of achieving tpCR. Accurate treatment plans are essential for healthcare providers, as they help reduce drug-related toxicity caused by excessive therapy and enable rapid modifications to treatment regimens.

In this study, variable reduction and selection conducted on the training set identified tumor size, Ki-67, molecular subtype, targeted therapy, and chemotherapy regimen as significant predictors of tpCR in NAC for LABC patients. Tumor size was shown to be a key independent factor in predicting tpCR post-NAC in patients with LABC. The finding is consistent with previous studies showing a strong correlation between smaller tumor size and a higher probability of obtaining tpCR 31, 32. The relation between this association may arise from the fact that patients with smaller tumors often have lower tumor burdens, less resistant cell populations, and are more responsive to chemotherapy. These factors combined contribute to improved results in NAC. Ki-67 levels, which are strongly associated with the rate at which tumor cells multiply, indicate the fast development and division of tumor cells. High levels of Ki-67 expression indicate that chemotherapy, which targets explicitly rapidly dividing cells, may be more successful. Therefore, Ki-67 is a significant indicator for predicting tpCR in BC patients post-NAC 33. However, the precise threshold for high Ki-67 expression remains debatable. The present study found a high expression threshold for Ki-67 at 47.5%, aligning with other research that set this threshold at around 50% 34. Further, due to the biological heterogeneity of BC, tpCR rates vary across molecular subtypes 35. Within the training set of the current study, 54.3% of patients who achieved tpCR had HER-2 positive BC, 32.5% had TNBC, and 13.2% had Luminal subtypes. This suggests that the rates of tpCR are greater in HER2-positive and TNBC subtypes compared to Luminal subtypes, which aligns with what has been reported in the previous research 36-39. Particularly, HER-2 positive BC patients receiving targeted therapy, such as trastuzumab and/or pertuzumab, had significantly improved tpCR rates 40, 41. Moreover, this study highlighted the chemotherapy regimen as a crucial characteristic for predicting tpCR, with anthracycline and taxane combinations being the most common NAC regimen 5. Compared to single-drug regimens, the joint application of these two types of drugs effectively reduces tumor cell resistance to medication. It generates a synergistic effect, enhancing cytotoxic activity against tumor cells. Similarly, incorporating platinum-based drugs, such as carboplatin, into the NAC regimen has been contentious; nevertheless, patients receiving platinum-based treatments have achieved higher tpCR rates 42, 43. In this study, patients treated with the anthracycline-taxane combination regimen exhibited significantly different tpCR rates than others. These variations may be strongly linked to a more accurate mix of chemotherapy drugs and immunotherapy and targeted treatment. Through the optimization of drug combinations and the full utilization of immunotherapy and targeted treatment approaches, a more thorough assault on tumor cells may be achieved, leading to enhanced therapeutic results and exhibiting a significant advantage in clinical practice. Building on this foundation, we developed and validated six ML models to predict the likelihood of pCR in patients with LABC undergoing NAC. After comprehensively comparing the performance of all models, we selected the KNN model, which was externally validated using an independent dataset from our hospital. The KNN model demonstrated high predictive accuracy across the training, testing, and external validation datasets, with AUC of 0.847, 0.763, and 0.665, respectively. In contrast, Kim et al. developed six ML models using 11 clinical and pathological features, with the LightGBM algorithm achieving the highest AUC of 0.810 7. Meti et al. selected seven clinical and pathological features to develop five ML models, with the RF algorithm achieving the highest AUC of 0.880 30. Our study's advantage lies in using only five clinical and pathological features while attaining comparable AUC values, demonstrating the model's simplicity and efficiency. This approach not only simplifies data collection and model training processes but also enhances the model's feasibility and scalability in practical applications. Furthermore, external validation confirmed the model's robustness and reliability, strengthening the credibility of our findings.

In contrast to other investigations, this research utilized SHAP technology to clarify the "black box" process of the ML model, improving its interpretability and therapeutic relevance. SHAP assessed the contribution of each predictive variable, with a SHAP importance plot visually presenting the ranking of variable contributions to the model's predictive capacity. The data indicated that targeted treatment was the most significant predictor for achieving a tpCR. Furthermore, individual waterfall plots for tpCR and non-tpCR patients not only augmented the model's interpretability but also provided clinicians with a more intuitive decision-support tool, greatly facilitating the practical use of predictive models in clinical settings.

It is imperative to acknowledge several unavoidable limitations inherent in this study. First, as a retrospective study, it is intrinsically prone to selection bias. Second, the external validation set consists of a restricted number of patients, and there was missing data for some variables. These factors have the potential to undermine the reliability and precision of model validation. Consequently, future research endeavors will necessitate large-scale, multi-center prospective studies for further validation. Moreover, the presence of spatial heterogeneity in tumors suggests that biopsy samples may not accurately reflect the entire tumor, thereby impacting the predictive precision of the model. Further, given the crucial role of radiological assessments in evaluating the efficacy of NAC 4, incorporating and integrating radiological data into future work will be a key step in optimizing the existing model. This approach will provide more precise diagnostic information and facilitate a more comprehensive evaluation of patient conditions and treatment outcomes, thereby better guiding clinical decision-making.

## Conclusions

This study successfully developed an ML predictive model based on the KNN algorithm, aimed at utilizing pre-NAC clinical pathology data to predict the probability of achieving a tpCR in patients with LABC at an early stage. This model incorporates several characteristics that are easily accessible before NAC therapy and utilizes SHAP technology to reveal the logical basis of its predictions, thereby tackling the difficulty of the "black box" problem that arises when implementing ML algorithms in clinical practice. The model is a dependable clinical prediction tool that helps doctors promptly modify treatment plans for patients not responding to NAC. This helps avoid missing out on the best surgical opportunities because of delays and encourages using more individualized treatment plans.

## Figures and Tables

**Table 1:** Baseline characteristics of patients with locally advanced breast cancer

Characteristics	Patients (N = 698)	Training set (N=468)	Test set (N=118)	External verification set (N=112)	*p-value*
Age, year					0.014
≤50	343 (49.1%)	248 (53.0%)	50 (42.4%)	45 (40.2%)	
>50	355 (50.9%)	220 (47.0%)	68 (57.6%)	67 (59.8%)	
Body mass index, kg/m^2^					0.407
≤23.52	346 (49.6%)	240 (51.3%)	53 (44.9%)	53 (47.3%)	
>23.52	352 (50.4%)	228 (48.7%)	65 (55.1%)	59 (52.7%)	
Menopausal status					0.129
Premenopause	355 (50.9%)	246 (52.6%)	50 (42.4%)	59 (52.7%)	
Postmenopause	343 (49.1%)	222 (47.4%)	68 (57.6%)	53 (47.3%)	
Axillary lymph node fusion					0.121
No	421 (71.8%)	343 (73.3%)	78 (66.1%)	0	
Yes	165 (28.2%)	125 (26.7%)	40 (33.9%)	0	
Chemotherapy regimen					0.001
Anthracycline combined with taxane	351 (50.3%)	269 (57.5%)	72 (61.0%)	88 (78.6%)	
Platinum	137 (19.6%)	102 (21.8%)	21 (17.8%)	14 (12.5%)	
Others	210 (30.1%)	97 (20.7%)	25 (21.2%)	10 (8.9%)	
Tumor size, cm					0.035
>3.14	442 (63.3%)	286 (61.1%)	73 (61.9%)	83 (74.1%)	
≤3.14	256 (36.7%)	182 (38.9%)	45 (38.1%)	29 (25.9%)	
Multifocality					0.167
No	492 (84.0%)	388 (82.9%)	104 (88.1%)	0	
Yes	94 (16.0%)	80 (17.1%)	14 (11.9%)	0	
Molecular subtype					0.826
Luminal	302 (43.2%)	198 (42.3%)	51 (43.2%)	53 (47.3%)	
HER-2 positive	237 (34.0%)	159 (34.0%)	40 (33.9%)	38 (33.9%)	
Triple negative	159 (22.8%)	111 (23.7%)	27 (22.9%)	21 (18.8%)	
Targeted therapy					0.484
No	484 (69.3%)	321 (68.6%)	80 (67.8%)	83 (74.1%)	
Yes	214 (30.7%)	147 (31.4%)	38 (32.2%)	29 (25.9%)	
Ki-67%					0.720
<47.5	381 (54.6%)	253 (54.1%)	63 (53.4%)	65 (58.0%)	
≥47.5	317 (45.4%)	215 (45.9%)	55 (46.6%)	47 (42.0%)	
Histological grade					0.886
Others	454 (77.5%)	362 (77.4%)	92 (78.0%)	0	
III	132 (22.5%)	106 (22.6%)	26 (22.0%)	0	
bpCR					0.383
No	456 (65.3%)	309 (66.0%)	80 (67.8%)	67 (59.8%)	
Yes	242 (34.7%)	159 (34.0%)	38 (32.2%)	45 (40.2%)	
tpCR					0.926
No	507 (72.6%)	339 (72.4%)	85 (72.0%)	83 (74.1%)	
Yes	191 (27.4%)	129 (27.6%)	33 (28.0%)	29 (25.9%)	

**Table 2:** Clinical and pathological characteristics according to tpCR in different sets.

Characteristics	Patients (N=698)	Training set (N=468)		*P value*	Test set (N=118)		*P value*	External verification set (N=112)		*P value*
		Non-tpCR	tpCR		Non-tpCR	tpCR		Non-tpCR	tpCR	
Age, year				0.366			0.673			.056
≤50	343(49.1%)	184 (54.3%)	64 (49.6%)		35 (41.2%)	15 (45.5%)		29 (34.9%)	16 (55.2%)	
>50	355(50.9%)	155 (45.7%)	65 (50.4%)		50 (58.8%)	18 (54.5%)		54 (65.1%)	13 (44.8%)	
Body mass index, kg/m2				0.861			0.190			.581
≤23.52	346 (49.6%)	173 (51.0%)	67 (51.9%)		35 (41.2%)	18 (54.5%)		38 (45.8%)	15 (51.7%)	
>23.52	352 (50.4%)	166 (49.0%)	62 (48.1%)		50 (58.8%)	15 (45.5%)		45 (54.2%)	14 (48.3%)	
Menopausal status				0.708			0.402			0.108
Premenopause	355 (50.9%)	180 (53.1%)	66 (51.2%)		34 (40.0%)	16 (48.5%)		40 (48.2%)	19 (65.5%)	
Postmenopause	343 (49.1%)	159 (46.9%)	63 (48.8%)		51 (60.0%)	17 (51.5%)		43 (51.8%)	10 (34.5%)	
Axillary lymph node fusion				0.718			0.037			
No	421 (71.8%)	250 (73.7%)	93 (72.1%)		61 (71.8%)	17 (51.5%)		0	0	
Yes	165 (28.2%)	89 (26.3%)	36 (27.9%)		24 (28.2%)	16 (48.5%)		0	0	
Chemotherapy regimen				<0.001			0.003			0.002
Anthracycline combined with taxane	351 (50.3%)	221 (65.2%)	48 (37.2%)		60 (70.6%)	12 (36.4%)		69 (83.2%)	19 (65.5%)	
Platinum	137 (19.6%)	59 (17.4%)	43 (33.3%)		12 (14.1%)	9 (27.2%)		5 (6.0%)	9 (31.0%)	
Others	210 (30.1%)	59 (17.4%)	38 (29.5%)		13 (15.3%)	12 (36.4%)		9 (10.8%)	1 (3.5%)	
Tumor size, cm				<0.001			0.805			0.217
>3.14	442 (63.3%)	226 (66.7%)	60 (46.5%)		52 (61.2%)	21 (63.6%)		59 (71.1%)	24 (82.8%)	
≤3.14	256 (36.7%)	113 (33.3%)	69 (53.5%)		33 (38.8%)	12 (36.4%)		24 (28.9%)	5 (17.2%)	
Multifocality				0.989			0.957			
No	492 (84.0%)	281 (82.9%)	107 (82.9%)		75 (88.2%)	29 (87.9%)		0	0	
Yes	94 (16.0%)	58 (17.1%)	22 (17.1%)		10 (11.8%)	4 (12.1%)		0	0	
Molecular subtype				<0.001			0.001			0.004
Luminal	302 (43.2%)	181 (53.4%)	17 (13.2%)		46 (54.1%)	5 (15.2%)		47 (56.6%)	6 (20.7%)	
HER-2 positive	237 (34.0%)	89 (26.3%)	70 (54.3%)		24 (28.2%)	16 (48.4%)		23 (27.7%)	15 (51.7%)	
Triple negative	159 (22.8%)	69 (20.3%)	42 (32.5%)		15 (17.7%)	12 (36.4%)		13 (15.7%)	8 (27.6%)	
Targeted therapy				<0.001						0.086
No	484 (69.3%)	259 (76.4%)	62 (48.1%)		63 (74.1%)	17 (51.5%)	0.018	65 (78.3%)	18 (62.1%)	
Yes	214 (30.7%)	80 (23.6%)	67 (51.9%)		22 (25.9%)	16 (48.5%)		18 (21.7%)	11 (37.9%)	
Ki-67%				<0.001			0.137			0.003
<47.5	381 (54.6%)	203 (59.9%)	50 (38.8%)		49 (57.6%)	14 (42.4%)		55 (66.3%)	10 (34.5%)	
≥47.5	317 (45.4%)	136 (40.1%)	79 (61.2%)		36 (42.4%)	19 (57.6%)		28 (33.7%)	19 (65.5%)	
Histological grade				0.054			0.177			
Others	454 (77.5%)	270 (79.6%)	92 (71.3%)		69 (81.2%)	23 (69.7%)		0	0	
III	132 (22.5%)	69 (20.4%)	37 (28.7%)		16 (18.8%)	10 (30.3%)		0	0	

*Statistically significant: p-value < 0.05.

**Table 3:** Predictive performance of six machine learning models

Subgroup	Model	Accuracy	F1 score	Kappa	precision	recall	PR_AUC	ROC_AUC (95%CI)	Brier score
Training set	KNN	0.741	0.632	0.447	0.520	0.806	0.693	0.847 (0.810, 0.883)	0.135
	LightGBM	0.701	0.600	0.386	0.475	0.814	0.578	0.801 (0.760, 0.842)	0.156
	DT	0.626	0.561	0.300	0.415	0.868	0.587	0.754 (0.708, 0.800)	0.163
	XGBoost	0.694	0.595	0.377	0.469	0.814	0.578	0.801 (0.760, 0.842)	0.161
	SVM	0.694	0.602	0.384	0.470	0.837	0.573	0.800 (0.759, 0.841)	0.163
	LR	0.701	0.600	0.386	0.475	0.814	0.573	0.796 (0.754, 0.837)	0.155
Test set	KNN	0.712	0.595	0.387	0.490	0.758	0.465	0.763 (0.670, 0.856)	0.182
	LightGBM	0.729	0.610	0.414	0.510	0.758	0.423	0.745 (0.653, 0.836)	0.178
	DT	0.627	0.560	0.296	0.418	0.848	0.448	0.686 (0.591, 0.780)	0.188
	XGBoost	0.644	0.543	0.288	0.424	0.758	0.429	0.741 (0.647, 0.834)	0.183
	SVM	0.686	0.565	0.338	0.462	0.727	0.415	0.737 (0.645, 0.828)	0.184
	LR	0.729	0.610	0.414	0.510	0.758	0.425	0.744 (0.652, 0.836)	0.181
